# Non-invasive focal therapy of organ confined prostate cancer with magnetic resonance guided focused ultrasound surgery

**DOI:** 10.1186/2050-5736-3-S1-P84

**Published:** 2015-06-30

**Authors:** Alessandro Napoli, Fulvio Zaccagna, Pier Luigi Di Paolo, Francesco Sandolo, Carola Palla, Fabrizio Andrani, Carlo Catalano

**Affiliations:** 1 University of Rome – Sapienza, Rome, Italy

## Background/introduction

To assess safety and effectiveness of non-invasive high intensity 3T MR guided Focused Ultrasound Surgery (MRgFUS) treatment of localized prostate cancer as salvage therapy and as an alternative to active surveillance.

## Methods

14 patients with biopsy proven focal T2 prostate cancer (low-to-intermediate risk: PSA max 12 and Gleason max 3+4), confirmed on a previous multiparametric MR exam (Discovery 750, GE) including dynamic contrast enhanced (DCE) imaging (Gd-BOPTA, Bracco), underwent MRgFUS ablation (ExAblate, InSightec). 1 patient had already performed radiotherapy without clinical success, 1 patient underwent US-guided Focused Ultrasound treatment with residual tumour tissue inside the gland needing a second treatment; the last 2 enrolled patients were part of an active surveillance regimen. All patients refused radical laparoscopic prostatectomy. MRgFUS treatment was carried out on the MR identifiable lesion (max 2) using a patient specific energy (3000-8500 J) and real time MR thermometry monitor for correct treatment location. Non-perfused volume (NPV) in the post-ablative MRI was used for necrosis assessment.

## Results and conclusions

Results: No significant complications were observed in all subjects during or immediately after the procedure. Post-treatment MRI demonstrated extensive coagulative necrosis at the site of sonication. At follow-up examinations 3 patients were free of residual viable tumor within the treated area; in the remaining patient, 10% of residual tumor was observed within the NPV. There was a variable amount of isolated cancer tissue (Gleason max 7, 3+4) within the non-treated parenchyma that was neither identifiable at MRI nor at biopsy.

Conclusion: Our results suggest that MR guided Focused Ultrasound Surgery is a safe and effective modality to determine >90% necrosis of identifiable prostate cancer; other prospective studies are needed to extend success rate in larger cohort.

